# Authentication of the Botanical and Geographical Origin and Detection of Adulteration of Olive Oil Using Gas Chromatography, Infrared and Raman Spectroscopy Techniques: A Review

**DOI:** 10.3390/foods10071565

**Published:** 2021-07-06

**Authors:** Eleni Kakouri, Panagiota-Kyriaki Revelou, Charalabos Kanakis, Dimitra Daferera, Christos S. Pappas, Petros A. Tarantilis

**Affiliations:** Laboratory of Chemistry, Department of Food Science and Human Nutrition, School of Food and Nutritional Sciences, Agricultural University of Athens, 111855 Athens, Greece; elenikakouri@aua.gr (E.K.); p.revelou@aua.gr (P.-K.R.); chkanakis@aua.gr (C.K.); daferera@aua.gr (D.D.); chrispap@aua.gr (C.S.P.)

**Keywords:** olive oil, authentication, adulteration, volatiles, IR, raman, gas chromatography

## Abstract

Olive oil is among the most popular supplements of the Mediterranean diet due to its high nutritional value. However, at the same time, because of economical purposes, it is also one of the products most subjected to adulteration. As a result, authenticity is an important issue of concern among authorities. Many analytical techniques, able to detect adulteration of olive oil, to identify its geographical and botanical origin and consequently guarantee its quality and authenticity, have been developed. This review paper discusses the use of infrared and Raman spectroscopy as candidate tools to examine the authenticity of olive oils. It also considers the volatile fraction as a marker to distinguish between different varieties and adulterated olive oils, using SPME combined with gas chromatography technique.

## 1. Introduction

According to the International Olive Council (IOC), olive oil can be defined as the product of the fruits from *Olea europea* L., trees obtained physically or mechanically by washing, decantation, centrifugation and filtration. These processes alter neither the nutritional composition of the final product nor interfere with its organoleptic characteristics, namely, taste and aroma. Regulation of the European Union (Reg. (EEC) 2568/91) and IOC have introduced specific requirements, according to which olive oil is classified in different categories which (a) define its quality, namely: alkyl esters of fatty acids composition (which are constituents connected to quality decline when their estimated value is greater than those imposed by legislative authorities), degree of acidity (expressed as a percentage of g of oleic acid in 100 g of oil), determination of peroxides (expressed as milliequivalents of active oxygen per kilogram which oxidize potassium iodide), spectrophotometric indices and sensory characteristics (flavor and aroma) and (b) are characteristic parameters of its purity such as quantification of waxes (the limit is set at 150 mg/kg for EVOO and VOO), sterols (total sterol content (mg/kg) as well as % of free sterols), 2-glyceryl mono-palmitate [1]. This classification includes Extra Virgin Olive Oil (EVOO), which is directly produced from olives using mechanical means. EVOOs’ aroma and taste are attributed to the presence of volatile and non-volatile compounds and their free acidity is lower than 0.8%. Virgin Olive Oil (VOO) is produced similarly to EVOO; however, its free acidity is higher but not greater than 2%, and its organoleptic properties are of inferior quality with respect to EVOO. Olive Oil (OO), is a mixture of refined and edible virgin olive oil, with acidity lower than 1%, and it is often labeled as virgin, light or pure olive oil. Refined olive oil is obtained from lower quality VOOs in terms of the degree of acidity and organoleptic characteristics. However, during refining processes such as deacidification, deodorization and decolorization, which require the use of organic solvents, these “defects” are usually eliminated. In particular, deodorization aims at removing off-flavor compounds from VOO, among which volatile compounds are included, that are produced via degradation/oxidation pathways due to long/inappropriate storage of olives before processing and/or of VOO. Deodorization requires high temperature under vacuum, which gradually degrades those unpleasant volatile compounds. Ordinary virgin oil is produced from olive oil fruits using the same processes as for EVOO; however, its acidity is 3.3%. This category has been excluded by the EU classification of olive oil, a strategy that will also follow other regulatory organizations. Lampante virgin olive oil is produced from low-quality fruits and is not destined for consumption. Its level of acidity is higher than 2%, and its organoleptic characteristics are rather poor (displeasing taste and smell). Finally, a distinct category that includes three subcategories is that kind of oil derived from pomace. Pomace is the residue of olive oil fruits after mechanical extraction of EVOO, which is treated with organic solvents, most common of which is hexane. Pomace oils include crude olive pomace oil, which is not adequate for human consumption; refined olive pomace oil, which is also not proposed for consumption and olive pomace oil, derived from olive pomace and VOO and is considered ideal for culinary purposes.

EVOO is a mixture of numerous bioactive compounds, the majority of which belong to monounsaturated fatty acids (MUFAs) such as oleic acid (18:1, n-9) and palmitoleic acid (16:1, n-7), with the first one being presented in abundance, and polyunsaturated fatty acids (PUFAs) such as linoleic acid (18:2, n-6) and linolenic acid (18:3, n-3). Other compounds such as vitamins (tocopherols), pigments such as β-carotene and lutein, phenolic compounds such as secoiridoids (derivatives based on hydroxytyrosol and tyrosol structures) and flavones (apigenin, luteolin) and hydrocarbons such as squalene, a terpenoid hydrocarbon and precursor of the biosynthesis of steroids, are found in minor quantity; however, they are endowed with important biologic activity [2,3]. Consequently, due to its rich composition in nutrients of unique nutritional value, many health claims have been made for its health-promoting properties [4,5,6]. Hence, a steady and rapid increase regarding its demand, inside and outside the Mediterranean basin, is observed. However, the high nutritional value, in combination with high production costs and high demand, makes olive oil a targeted product fraud. Therefore, economically motivated adulteration incidents and cases of fraudulence are continuously revealed.

In order to avoid any kind of adulteration, the olive oil market is closely monitored by various organizations, which have suggested criteria and limits that should be respected by producers and distributors. Quality criteria including chemical composition parameters, organoleptic characteristics (aroma, taste, flavor), and guidelines of analysis methods have been proposed by the IOC and the Codex Alimentarius (CODEX STAN 33-1981). These proposed standards are not mandatory; however, they should be respected by the countries that agree to follow them. On the other hand, regulation of the European Community (EEC) No 2568/91 imposes compulsory parameters regarding quality and purity criteria, that should be adopted by the EU members. Furthermore, with the European Union regulation No 29/2012, geographic-origin labeling becomes a prerequisite.

The authenticity of EVOO and VOO is of major importance for both financial and health-related reasons. To this end, various analytical techniques, able to detect possible adulteration and consequently ensure its quality and authenticity, have been developed. Among them, spectroscopic techniques such as Nuclear magnetic resonance (NMR), infrared (near, mid, far) and Raman spectroscopy and chromatographic methods such as liquid and gas chromatography have been used by various researchers to determine qualitative and quantitative changes in the composition of EVOO and VOO that are related to adulteration issues [7,8,9,10]. Among the techniques mentioned above, gas chromatography is the most used technique regarding authentication issues that deal with geographical and botanical discrimination, while infrared and Raman spectroscopy are commonly used in cases of adulteration detection, as both are less time-consuming techniques and require no sample preparation.

Due to the complex nature of the obtained data, manipulation with statistical methods so as to obtain a final report should be followed. In this regard, multivariate analysis has been proven to be a valuable tool. Many studies have pointed out the usefulness of this method and support its use in many sciences such as chemistry, health sciences and analytical chemistry [11,12,13,14].

The goal of this review paper is to provide information over the last 20 years about olive oil authentication. Specifically, the review focuses on EVOO and VOO, the most consumed types of olive oil, and describes rapid techniques able to accurately predict their authentication. Articles regarding Infrared (IR) and Raman spectroscopy as well as gas chromatography (GC), able to detect adulteration and to classify EVOO and VOO according to their geographical and botanical origin, are presented by giving a brief summary of the vast available published information.

## 2. Spectroscopic Techniques Used to Evaluate Adulteration of EVOO and VOO: Focus on Infrared and Raman Spectroscopy

IR spectroscopy is a fundamental and popular method for the qualitative and quantitative analysis of complex matrices. The information generated is attributed to the presence of characteristic groups such as alcohols, ketones, alkenes, etc. The obtained spectrum is unique for each tested sample and represents a distinctive chemical fingerprint. Raman spectroscopy, such as with IR, provides a unique spectrum for each sample. However, the difference between these two techniques lays in the characteristic absorption bands. Unlike IR, intense and well-distinctive bands of symmetric vibrations of non-polar groups appear in Raman spectroscopy, thus making able to determine, for example, the type of unsaturated fatty acids and ensure the presence of cis configuration. Both techniques are quick since no preparation of the sample is required; consequently, the use of solvents is limited to the minimum and the sample remains intact. Time of analysis is short and information obtained, when combine results, complete the profile of the studied sample and allow researchers to guarantee its authenticity [15] (Table 1).

Azinian et al. (2015) [7], using Near-Infrared Spectroscopy (NIRS), developed a novel protocol to confirm the authenticity and adulteration of EVOO based on the creation of an FT-NIR index, determined by specific wavelengths. Authenticity was achieved thanks to two characteristic absorption bands, at 5180 cm^−1^, which corresponds to non-volatile compounds or esters and at 5280 cm^−1^, which equals to volatile compounds bearing carbonyl groups. Detection of adulteration was evaluated based on the spectral differences between the fatty acid (FA) composition among EVOO and adulterant oil. In cases of similarities between the FA composition of EVOO and the adulterant, a higher level of adulteration was necessary. According to the FT-NIR index, a classification model using Partial Least Square Regression analysis (PLS) was built, and results evidenced that the method developed by the authors is of high reliability. A similar study analyzed commercial samples of EVOO for their authentication with the FT-NIR technique [10]. Authors’ prerequisites in order to define a sample as EVOO were an FT-NIR index greater than 75%, the content of the main FA was according to official criteria guidelines and the adulterant content at the level of limit ±2SD (standard deviation), as established by other studies [7]. Principal Component analysis (PCA) and PLS analysis classified 33 of the 88 samples labeled as EVOO according to national authorities since adulteration with oils of minor quality was detected.

Mixing EVOO with hazelnut oil is a common type of fraud. These two types of oil have similar chemical compositions; as a result, detection of low percentages of the adulterant is quite difficult. Raman spectroscopy was used by López-Díez et al. (2003) [16], who distinguished EVOO of similar cultivars and quantified the degree of adulteration with refined hazelnut oil, using PCA analysis and PLS analysis in order to simplify data and to quantify adulteration. Results showed that quantification of adulteration was achieved to a range between 0 and 20%. Likewise, Davies et al. (2000) [17] determined adulteration of greek EVOOs with sunflower oil. Fourier transformed (FT-NIR) Raman spectroscopy was used, and PCA analysis confirmed the addition of sunflower oil at a level of 2% due to clear spectrum differences at the 1300–1250 cm^−1^ region. Following the good classification rates regarding PCA analysis, the authors examined whether the PLS method also provides satisfactory results. A full spectrum analysis was first performed without choosing a specific wavenumbers area. By restricting the samples used in one geographic area, the root means square error of prediction (RMSEP) significantly improved (1.69% instead of 2.86% in the first case). Additional tests included a selection of a specific wavelength and comparison of the root means square error of calibration (RMSEC) and RMSEP values between samples from different geographic regions and samples from the same region. According to their results, the less the geographic variability of the selected samples, the better the predictive capacity of the model. In another study, Vis-Raman was used to classify vegetable oils and to detect adulteration of EVOO. Discrimination between EVOO and vegetable oils, as well as detection of adulterated EVOO with sunflower oil, were performed with the PCA analysis in combination with multidimensional scaling (MDS). Adulteration was detectable at levels lower than 5%. Afterward, PLS analysis was used to design the calibration model and to quantify the adulterant. Spectral regions according to which the PLS model was designed were 800–1800 and 3020–2850 cm^−1^ [18]. In a similar study, adulteration of Greek EVOO with sunflower oil was investigated by Phillipidis et al. (2016) [19], using Raman and visible spectroscopy. Regarding Raman spectroscopy, PLS method and a three latent variable algorithm were used to distinguish between EVOO and the adulterant. Characteristic peaks at 1265 and 1657 cm^−1^, which belong to monounsaturated and polyunsaturated fatty acids, respectively, are more intense in the case of sunflower oil. In addition to the above-mentioned characteristic peaks, absorption bands at 1082 and 1657 cm^−1^ were used for sufficient differentiation. Model robustness was evaluated by cross- and external validation, using the R^2^ and root mean square error of cross-validation (RMSECV) parameters in the first case, while for the second one, the RMSEP was examined. In addition, the authors used a Raman spectrometer with a visible laser to examine the presence of carotenoids presented at 1004, 1156 and 1527 cm^−1^, which appear only at the samples of EVOO. Furthermore, adulteration of EVOO with 5% soyabean oil using Raman spectroscopy combined with Linear Discriminant Analysis (LDA) was also evaluated by Ryoo et al., 2017 [20]. PCA analysis was selected to diminish data variability, and LDA analysis followed. However, their experiment differed from other studies in which authors supported that since compounds of an EVOO are not structurally similar; the selection of an optimal temperature that enhances authentication accuracy is of critical importance. Their assumption was evaluated based on spectral differences acquired at different temperatures, using as markers linoleic and oleic acids. In another study by Jiménez-Carvelo et al. (2017) [21], both Fourier transform infrared (FT-IR) and Raman spectroscopy were used to distinguish pure olive oil from those mixed with edible oils, using multivariate methods. Among the classification models used, Partial Least-Squares Discriminant analysis (PLS-DA) model gave the best results regarding FT-IR, while for Raman spectroscopy, the Support Vector Machine using parameter C (SVM-C) model was found to be more reliable. In addition, quantification of adulteration, where various amounts of adulterant were examined, was performed using the Partial Least Squares Regression (PLS-R) model. Raman gave better results regarding the R^2^ value; on the contrary, the value of quantification error was better when evaluated with the FT-IR. The authors described the developed method as a “global method” that allows “detection, discrimination and quantification” of virgin olive oil, pure or adulterated. A preliminary step of transesterification was needed prior to analysis.

Ozen et al. (2002) [22] and Maggio et al. (2010) [23] used FT-IR spectroscopy in combination with chemometrics (Discriminant Analysis (DA) and PLS) to evaluate adulteration of EVOO with hazelnut and other oils of minor quality. Both studies concluded that FT-IR could be used as an alternative, quick and economic method for the detection of hazelnut oil. However, Ozen et al. (2002) [22] showed that although classification between these two types was successfully achieved, adulteration was only detected at levels higher than 25%. Rohman et al. (2010) [24] studied the adulteration of EVOO with various vegetable oils, including palm oil, using FT-IR spectroscopy. DA was performed in order to classify pure and adulterated EVOO. Quantification of the adulterant was performed in EVOO-palm oil mixtures using PLS and PCA models. The obtained results were satisfactory, taking into account the calculated values derived from the models applied that correspond to R^2^ equal to 0.999 for both models and RMSECV values equal to 0.285 and 0.373, respectively. The 1500–1000 cm^−1^ fingerprint area was chosen to build the chemometric models. In another study, adulteration of EVOO with sunflower and soybean oil was determined with mid-IR spectroscopy. The authors used the PLS and PLS-DA methods to quantify adulteration at a range of 1–24% weight ratios. They focused on the 3035–2800 cm^−1^, 1800–1700 cm^−1^ and 1400–670 cm^−1^ spectral regions for data interpretation. However, in order to reduce the number of the selected variables, the Variable Importance of Projection (VIP) method was performed, which permitted a reduction of approximately 75% of the variables used. Based on R^2^, RMSEC, RMSECV and bias values, a strong correlation between the true and predicted values was achieved, confirming that the PLS-VIP method can be successfully used. In addition, a 100% correct classification of the samples was achieved with the PLS-DA analysis [25].

Mid-infrared spectroscopy (MIR) was used by Hirri et al. (2015) [26] in order to detect adulteration of EVOO with lampante virgin oil. Two calibration models were constructed, using PLS and PCR analysis, according to which quantification of the adulterant was achieved. 

Vanstone et al. (2018), [27] examined several cases of adulteration using NIR. First, they tested the capacity of the method to distinguish between a known sample of EVOO (control) adulterated with corn oil (2.7–25% *w/w*). A good separation between EVOO samples, pure and adulterated, was achieved using the PCA method. In particular, the greater the degree of adulteration, the more major the differences between the tested samples. The authors also examined the case in which NIR spectroscopy could detect the adulteration of unknown EVOO samples. They used in total 37 EVOO samples (separated into 27 calibration and 10 validation models) with different qualitative characteristics, cost, degree of oxidation and fatty acid profile. Validation samples were mixed with various adulterants, including corn, sunflower, canola and soyabean oils. Adulterants were detectable with NIR spectroscopy; however, limits of detection were higher than 2.7%. Specifically for corn and sunflower oils, the adulteration limit was 20%, while for canola and soyabean oil, it was 10 and 15%, respectively. The fact that the chemical and physical characteristics of an EVOO depend on various parameters such as geographic origin and climate conditions, which consequently influence its chemical profile, including volatile and fatty acid constituents, make more complicate the detection of adulteration, especially when no control sample is provided. Nevertheless, the authors claimed that based on fatty acid content and specifically on palmitic, stearic, oleic, linoleic and linolenic acids, if limits of quantification are precisely determined, adulteration is detectable. However, usually, this is achieved by adding a relatively high percentage of the adulterant. EVOO mixed with corn and sunflower oil were correctly classified as pure or not using PCA and soft dependent modeling of class analogies (SIMCA) classification method. Similarly, adulteration of EVOO with canola oil or soybean oil was studied. In this case, observations were focused on the fatty acid composition and specifically on linoleic acid (LA). The level of this fatty acid is higher at the adulterated agent with respect to EVOO; thus, LA is considered as a candidate marker to distinguish between EVOO and minor quality olive oil. NIR spectroscopy was used by Jiménez-Carvelo et al. (2019) [28] in combination with PCA and PLS1-DA. The authors managed to adequately classify (100%) Argentinean EVOOs according to their geographical origin when NIR was combined with PLS1-DA analysis and also detect adulteration with oils of minor quality. Jiang and Chen (2019) [29] used FT-NIR in combination with bootstrapping soft shrinkage (BOSS) algorithm and PLS analysis to successfully detect adulteration of EVOO with a variety of adulterants, including peanut, sunflower seed oil, soybean, sesame and corn oil. Adulteration was detected and quantified at a level of 5–50%. The authors used different algorithms in order to select the most appropriate wavenumbers, such as competitive adaptive reweighted sampling (CARS), Monte Carlo uninformative variable elimination (MCUVE) and iteratively retaining informative variables (IRIV); however, they concluded that the best algorithm to use is the BOSS fitted in the PLS model, as it gave the best value of R^2^ (0.99) and the lowest RMSECV value (1.44%). NIR, Mid-infrared spectroscopy (MIR) and Raman spectroscopy were compared in terms of their capacity to predict adulteration of EVOO with soybean oil. In addition, the fatty acid composition of EVOO and soybean was first analyzed with GC-FID. Authors observed that palmitoleic acid (POA) was only found in the EVOO samples; thus, this compound was suggested as a label to quantify adulteration. However, on the contrary to GC-FID analysis, POA is evident in both EVOO and soybean oil at Raman spectroscopy. Nonetheless, quantitative differences of LA were observed, and thus LA was used for the quantification in combination with PLS analysis. The full spectrum was used to analyze the results. Although RMSECV and RMSEP values were higher when MIR spectroscopy was used, the authors concluded that all the methods performed are suitable for detecting EVOO adulteration. This statement was also confirmed by a *t*-test which showed no statistical differences between the predicted values and the true ones [30]. Similarly, MIR and NIR spectroscopy were used to detect adulteration of EVOO with refined and deodorized oils. The spectral region finally chosen by the authors was 1500–900 cm^−1^ and 6150–4500 cm^−1^ for MIR and NIR, respectively. Both techniques were combined with PLS and PCR analysis, and the best cross-validation results according to R^2^ and RMSECV values were obtained with the MIR-PLS model (R^2^ = 0.98, RMSECV = 2.9) [31].

FT-NIR spectroscopy was also applied by Karunathilaka et al. (2016) [32], who classified OO samples (labeled as EVOO and non-EVOO), using the univariate conformity index (CI) and the SIMCA model. As reference and training sets for the CI and SIMCA models, monovarietal and oil from different varieties were used. Spectra were recorded at 4000–1250 cm^−1^ while significant spectral differences were observed at the region 9002–4552 cm^−1^ when the CI model was applied and at the region 5350–5150 cm^−1^, when the SIMCA model was used. Better discrimination results were obtained with the SIMCA model. Similarly, another research team tried to distinguish EVOO from lower-quality oils using the Handheld NIRS Analysis. A PCA analysis was first performed to select the most adequate variables, and the spectral region chosen by the authors based on characteristic spectral differences between the studied samples was 1570–1350 cm^−1^. Various classification models were built, including one-class k-nearest neighbors (OCkNN), one-class support vector machine (OCSVM) and SIMCA. The sensitivity, accuracy and specificity of the SIMCA model was 100%, followed by the OCkNN model [33].

Tay et al. (2002) [34] studied the authenticity of EVOO using the Attenuated Total Reflection (ATR) technique. Authors used DA, and specifically, the 3100–2800 cm^−1^ and 1800–900 cm^−1^ spectral regions, and 100% discrimination between the samples of EVOO adulterated or pure was achieved at 20 mL/L of the adulterant. Quantification of sunflower was performed by the PLS analysis. Similar results were also obtained by Filoda et al. (2019) [35] by also using the ATR technique and PLS analysis. The spectral region studied was between 3200–650 cm^−1,^ and a unique prediction model was constructed, able to detect adulteration of EVOO with refined soybean oil, refined sunflower oil, refined corn oil and refined canola oil, independent of the adulterant and the degree of adulteration.

### Spectroscopic Techniques Used to Evaluate the Authenticity of EVOO and VOO According to Their Variety and Geographical and Botanical Origin: Focus on Infrared and Raman Spectroscopy

Raman spectroscopy was used for the geographical discrimination of EVOO from Italy on a minor number of samples (38 samples). In this study, isotope ratio mass spectrometry (IRMS) and resonant Raman spectroscopy (RRS) were used and the development of the chemometric model was based on LDA. Correct classification rate was 82% [36]. Moreover, Benlamaalam et al. (2015) [37] classified 49 samples of EVOO from different areas of Morocco using MIR spectroscopy in combination with PCA and PLS-DA analysis. Similarly, de Luca et al. 2011 [38] classified Morocco VOOs with MIR spectroscopic analysis. First, using cluster analysis (CA) in combination with the average paired distance algorithm, samples were divided into four clusters according to their geographical origin; classification with CA was further certified with PCA analysis. Additionally, authors classified EVOO samples according to their harvest time, but from the same geographic area using PLS-DA. For the optimal selection of the wavelengths to include in the classification model, Martens’ Uncertainty test was performed, which led to a better R^2^ and RMSEP value, with respect to the analysis performed prior wavelength selection. Downey et al. (2003), [39] used the NIR technique to distinguish between cultivars of EVOO produced in nearby regions. They performed a PLS, factorial discriminant analysis (FDA) and k-nearest neighbor analysis (k-NN). FDA gave the best classification rate; however, all three models revealed that NIR is a powerful candidate method to use when determining the geographical origin of EVOOs. Geographical and botanical discrimination of 412 Andalusian EVOOs was achieved with Raman spectroscopy, based on MUFA, saturated fatty acids (SAFA) and PUFAs presence. PLS analysis was used to select the most appropriate variables, and using DA, high correct classification rates of the samples according to their place of origin, year of harvest, Protected Designation of Origin (PDO) and variety were achieved (89, 94.3, 86.6 and 84%, respectively) [40]. Furthermore, FT-NIR spectroscopy was applied to separate Tunisian VOOs that belonged to six different cultivars using the PCA analysis. The classification was achieved by the SIMCA method [41].

In a similar study, discrimination of EVOOs of different varieties was also achieved by NIR and MIR spectroscopy. Results were analyzed with the LDA and SIMCA methods, while the spectral region used was chosen by the SELECT algorithm. The best results were obtained by MIR spectroscopy, in which classification and prediction rates were 94.2 and 86.6%, respectively [42].

In addition, Bendini et al. (2007) [43] analyzed 84 samples of EVOO derived from different regions of Italy and classified them according to their geographical origin using ATR-FTIR combined with PLS and PCA analysis. Only the fingerprint zone of the spectra was used to classify samples, and that is, according to the authors, the region between 1720–700 cm^−1^. Most of the samples were correctly classified, while for some, moderate classification was achieved, probably, as stated by the authors, because of the small number of the samples or due to lack of genetic variation between them. Botanical discrimination of Greek cultivars was achieved using ATR-FTIR combined with chemometric methods. The forward stepwise selection algorithm was chosen for the selection of the spectral region that discriminated the olive oil samples, followed by the application of the algorithms LDA and quadratic discriminant analysis (QDA) analysis. The classification was achieved for both chemometric models [44]. The geographical origin of 60 EVOOs was also studied by Tapp et al. (2003) [45] using FT-IR and samples of different European countries. They developed two models of data analysis using PLS followed by LDA. After they combined LDA with a genetic algorithm (GA) based on the selection of eight varieties according to the first analysis, which generated a good prediction model. The second model gave better results regarding the cross-validation rate (100%), contrary to the first (96%).

## 3. Detection of EVOO and VOO Adulteration by Gas Chromatography

A common technique used by many researchers is solid-phase microextraction (SPME) accompanied with gas chromatography, which allows the characterization and quantification of the volatile composition of olive oil. SPME is a routine, low-cost, easy and efficient technique developed by Arthur and Pawlszyn in 1990 [46]. The technique occupies the use of a fiber coated with an extracting phase which can be solid or liquid, able to extract volatile and semi-volatile compounds from a sample. The sample is exposed to the fiber under steady temperature and stirred for a certain time, until the extracted compounds are absorbed by the fiber to the maximum of their concentration. Then, the SPME fiber can be directly injected into the GC system, where helium carries the volatile compounds into the capillary column and analysis of the sample begins (Table 2).

Flores et al. (2006) [47] studied the adulteration of EVOO with hazelnut oil using various techniques based on its volatile fraction, among which SPME-multidimensional gas chromatography (SPME-MDGC). For the experiment, a Divinylbenzene/Polydimethylsiloxane (PDMS/DVB) fiber was used. Authors preferred to label an EVOO as adulterated only when both R and S enantiomers of filbertone were identified after an analysis of the results. Detection of adulteration was achieved to a range of 7–25% for hazelnut oil. One of the most important findings of this study was the fact that the hazelnut oil used was derived from unroasted raw material. This is important because levels of filbertone in this type of oil are lower than those derived from roasted hazelnuts oils [63]. Mildner-Szkudlarz et al. (2008) [48], using SPME/gas chromatography combined with mass spectrometry (GC-MS) and PCA, showed that gas chromatography could detect adulteration of an EVOO with hazelnut oil at 5% (*v/v*). Chemically, the volatile profile of these two oils is different. For example, EVOO is characterized by a high level of aldehydes (68%), whereas in olive oil of minor quality, the respective percentage is lower (38%), while ketones and acids constitute the majority. In this study, compounds that belong to the aldehydes family, as well as other compounds, helped distinguish EVOO from hazelnut oil, and these include E-2-hexenal, hexanal, pentanal, acetic acid, 2-octanone and 2-heptanone. Furthermore, many compounds such as propanoic acid, toluene and butanoic acid are present only in hazelnut oil. Consequently, based on quantitative and qualitative differences of the chemical profile between EVOO and hazelnut oil and by using chemometrics, adulteration can be detected.

### 3.1. Volatile Profile of EVOO and VOO as an Index for Evaluating Its Authenticity by Gas Chromatography Based on Geographical Origin

Olive oil volatile profile was studied by Cajka et al. (2004) [49], who aimed at distinguishing between EVOOs of different geographical origins. They used headspace (HS)-SPME followed by gas chromatography-ion trap mass spectrometry (GC-ITMS) combined with PCA, LDA and an artificial neural network with multilayer perception (ANN-MLP). The authors tested various types of fibers for their efficacy; however, they concluded that the most suitable was the DVB/CAR/PDMS (50/30 μm) fiber. Samples were analyzed with the GC-ITMS technique, yet authors proposed that for those compounds where GC chromatographs were not clear, Two-Dimensional Gas Chromatography and Time-of-Flight Mass Spectrometry (GCXGC-TOFMS) was the adequate analysis to perform. Indeed, spectra quality was significantly improved. Regarding multivariate methods, hallmark compounds such as alcohols, aldehydes and esters were chosen in order to build the chemometric model. A PCA analysis was first performed, and afterward, the LDA analysis followed. Nevertheless, the last analysis was rather poor in classifying samples. In this regard, ANN-MLP analysis was performed, which definitely predicted an acceptable classification value. In another study performed by García-González et al. (2010) [50], monovarietal VOOs, produced in different regions of Chile, were distinguished based on their volatile and phenolic composition. Regarding volatile compounds, they were collected using the SPME method and a DVB/CAR/PDMS 50/30 μm fiber. Volatiles were separated with GC. Data were then handled with PCA and Analysis of Variance (ANOVA), and successful classification of the samples showed that climatic, cultivar and soil conditions are parameters that allow differentiation between monovarietal olive oil and determination of their geographical origin. In addition, the authors compared two varieties produced in Chile with the respective varieties produced in Spain. They concluded again that the volatile profile based on quantitation calculations is a marker that helps in distinguishing the country of provenance. Berlioz et al. (2006) [55] analyzed French virgin olive oils with PDO in order to confirm their authenticity by comparing their volatile profile with commercial EVOO. Volatiles were absorbed on a Divinylbenzene/Carboxen/Polydimethylsiloxane (DVB/CAR/PDMS) 50/30 μm fiber and then separated with the GC-FID technique. Results were first subjected to PCA analysis, where significant differences were observed between the PDO oils and the control ones. In particular, decreased amount of E-2-hexenal at the commercial EVOO was a key result that allowed discrimination between PDO and commercial oils. Finally, SIMCA analysis gave a good classification model as constructed by data based on particular compounds, characterized as marker compounds. A similar study was performed by Pizarro et al. (2011) [51], who related the geographical origin of EVOOs from various Spanish regions with the volatile profile. HS-SPME/GC-MS technique was performed and chemometric analysis, namely, PCA and Stepwise Linear Discriminant Analysis (SLDA), were used to explain results and classify the studied samples. Classification of the samples with the PCA analysis was not satisfactory; however, preliminary information regarding the formation of the classification model was obtained. On the other hand, six characteristic compounds were chosen in order to perform the SLDA analysis, and results showed a 100% discrimination rate among the different varieties. In another study, EVOOs from different countries were classified according to their geographical origin using the sesquiterpenes profile as a marker. Samples were analyzed with the HS-SPME-GC-MS technique with a DVB/CAR/PDMS 50/30 μm fiber, and PCA analysis was performed. The classification model was constructed with the PLS-DA analysis, and results were analyzed using both profiling and fingerprinting analytical approaches. The non-targeted approach gave better classification rates. In particular, all samples were 100% correctly classified, whereas the targeted approach varied from 46 to 100% [52]. Likewise, geographic and variety differentiation of VOOs was studied regarding Italian VOOs. SPME, GC-MS and gas chromatography with flame ionization detection (GC-FID) techniques were used to collect, identify and quantify, respectively, the results. Samples were characterized by the presence of C6 and C5 compounds, while other compounds such as cis copaene, cis farnesene presented significant quantitative differences between the varieties. Differences regarding the cultivars were studied with ANOVA analysis while DA combined with PCA was used for classification, based on their geographic region. A 100% correct classification rate was achieved [53]. Youssef et al. (2011) [54] also studied the influence of the geographical region on the aroma of VOOs from the same variety. The authors used HS-SPME, GC-MS and GC-FID methods to evaluate the chemical profile of the samples. Results showed that samples did not present significant differences regarding their qualitative profile; however, quantitative differences exist and helped differentiate VOOs according to their geographical origin with ANOVA, PCA and Hierarchical Cluster Analysis (HCA).

### 3.2. Volatile Profile of EVOO and VOO as an Index for Evaluating Its Authenticity by Gas Chromatography Based on Variety/Botanical Origin

Kaftan and Elmaci (2011) [56] studied the differences of the volatile compounds between two VOO varieties from Turkey (Ayvalik and Memecik) using the SPME/GC/MS technique. The fiber used was the PDMS/DVB/CAR 50/30 μm. Cluster and PCA analyses were performed to model results. Authors found that between these two varieties, qualitative and quantitative differences based on the presence of characteristic volatile compounds can lead to their differentiation. In particular, for the Ayvalik variety, hexanal, 3-hexen-1-ol, cis-3-hexenol and 9-octadecenoic acid were the discriminating compounds, whereas, for Memecik variety, 2-hexanal and 3-hexen-1-ol acetate were the most characteristic compounds.

Kosma et al. (2016) [57] studied the volatile and fatty acid profile of six cultivars of EVOO produced in Greece. The volatile profile was evaluated with the HS-SPME-GC/MS method using a DVB/CAR/PBMS 30/50 μm fiber. Compounds that belong to the C6 including (E)-3-hexen-1-ol, (E)-2-hexen-1-ol, 1-hexenol, (Z)-3-hexenal, hexanal, (E)-2-hexenal, (E, E)-2, 4-hexadienal and hexane. (E)-2-hexenal and C5, namely, 1-penten-3-ol, 1-pentanol, cis-2-pentenol, 1-penten-3-ne, 2-pentanone, 3-pentanone, pentanal, (E)-2-pentenal, pentane, (Z)-1,3-pentadiene and 3-methyl butanal group, as well as esters, hydrocarbons and terpenes were identified. According to Multivariate analysis of variance (MANOVA), 34 samples were chosen to perform the LDA analysis. Differentiation between cultivars was successfully achieved and presented a high classification rate which corresponds to 83%. Croatian VOOs were studied by Lukic et al. (2019) [58], who attempted to differentiate them according to their geographical and variety origin. HS-SPME technique was used to collect volatile compounds with the use of a DVB/CAR/PBMS fiber. Conventional mono-dimensional GC-MS in combination with GC-TOF-MS were used to separate the collected volatiles. Since TOF-MS is a technique known for its better selectivity and accuracy, more compounds that cannot be detected with the simple GC-MS were identified. In combination with chemometric analysis (ANOVA, PCA and SLDA), samples were successfully differentiated, whereas the presence of monoterpenes, sesquiterpenes and terpenes was determinant for their classification.

The volatile profile of six Algerian VOO varieties was studied by Nigri et al. (2012) [59]. HS-SPME (DVB/CAR/PBMS 50/30 μm fiber) followed by GC-MS was used to identify compounds. No chemometrics analysis was performed, and characterization of each variety was based on quantitative differences among the tested samples, which permitted their differentiation according to geographic criteria.

Mono and sesquiterpenes profiles of VOOs from different varieties derived from Spain and Italy were studied in order to determine differences that could lead to their differentiation [60]. The authors examined several parameters regarding the HS-SPME method, including the type of the fiber, extraction and temperature time, to find the best conditions which give the optimum chromatographic profile as derived from the GC-MS technique. They concluded that a DVB/CAR/PDMS fiber and the extraction temperature significantly affected the results obtained, on the contrary to the extraction time, which played no particular role. Quantitative differences of the studied compounds were more evident regarding sesquiterpenes, even though minor differences of the monoterpenes profile were also detected between some varieties. Taking into account only the mentioned quantitative differences, the authors supported that mono and sesquiterpenes can be a reliable index in distinguishing virgin olive oils based on cultivar and geographical parameters. In the study of Luna et al. (2005) [61], VOOs varieties from different countries but grown exactly under the same conditions (climate, irrigation, region) were examined in order to determine differences that could lead to their geographic identification. Dynamic headspace gas chromatography was used, and statistical analysis, including Brown–Forsythe test and SLDA was followed. Based on a strict number of compounds as derived from the Brown–Forsythe test, Spanish from Greek and Greek from Italian varieties were 100% correctly classified while between Spanish and Italian samples, the correct classification rate was 100% and 71.4%, respectively. Blasi et al. (2019) [9] classified EVOOs of different varieties according to their FA and volatile composition. In particular, regarding the volatile fraction, SPME/GC-MS method was used. Qualification and quantification of the compounds detected revealed that E-2-hexenal was the most abundant compound presented in all the studied varieties, followed by trans-2-hexen-1-ol. Based on the quantitative differences of these two compounds, successful classification of the samples according to their variety was achieved using the LDA analysis.

Pouliarekou et al. (2011) [62] examined VOOs from Western Greece. The SPME (DVB/CAR/PBMS 30/50 μm fiber) and GC/MS techniques were used to collect and separate volatile compounds. As mentioned above, in this study, most of the identified compounds belonged to the C6 and C5 family. ANOVA and LDA analysis were chosen for the classification of the results according to cultivar and geographical factors, and 74 and 87.2% correct classification rate, respectively, was achieved. Discrimination of the three most commercial Greek olive oil cultivars has also been achieved by SPME GC/MS in combination with the stepwise LDA and QDA algorithms. The correct classification rate reached 97.4 and 100%, respectively. The major volatile compounds selected for the discrimination of samples were terpenoid hydrocarbons [44].

## 4. Discussion

According to ISO 9000, quality is defined as “the degree to which a set of inherent characteristics of an object fulfills requirements”. These requirements are imposed via rules and regulations by official authorities in order to combat fraud problems. Regarding olive oil, and especially EVOO and to a lesser extend VOO, issues of authentication are a continuous concern for authorities. Authentication issue includes a check of possible adulteration of EVOO with other types of oil of lower value and minor quality, misleading geographical indication and deceptive botanical origin. The most common adulterants usually detected at EVOO and VOO are various vegetable oils of inferior economical value and different in compositional characteristics, such as canola, sunflower, hazelnut and soyabean oils.

Although regulations regarding olive oil quality have been proposed and limits of various physicochemical parameters have been imposed, the drawback of these recommendations is that they are not obligatory worldwide. In addition, the common harmonized EU law 2568/91 is mandatory for all EU members; however, as described by Conte et al. (2019) [64], still, minor improvement is needed. In these terms, the need for simple, rapid, environmentally friendly and reliable techniques able to confirm authentication of olive oil and consequently certify its quality is obligatory since safety is not negotiable for consumers.

As has been already mentioned, EVOO and VOO contain many active constituents that belong to different chemical families. Given the fact that the chemical profile is strongly affected by numerous factors (climate, soil, cultivar, storage, collection), it is obvious that a “standard” chemical profile of olive oil, based on its category according to IOC guidelines, is difficult to adopt. This complexity consists of a serious advantage when cases of fraudulence are planned, and at the same time, a major disadvantage for those organizations that combat fraud. Nevertheless, the development of many analytical techniques has considerably contributed to combatting the fraud apocalypse.

EVOO authenticity is guaranteed by its unique, peculiar aroma owned to the presence of multiple volatile compounds that belong to different chemical groups, including aldehydes, ketones, alcohols and esters [65,66,67]. Specifically, the presence of C6 and C5 aldehydes and alcohols as well as their esters, are characteristic compounds to which the sensory quality of EVOO is attributed. These compounds are produced by the lipoxygenase (LOX) pathway and are presented at high concentrations in EVOOs, on the contrary, lower quality oils are present in minor quantities or traces or are even absent [68]. Although the volatile composition of an olive oil strongly depends on climatic, geographical, cultivar and extraction methods, various analytical techniques have been developed, able to characterize the oil’s authenticity, variety and geographical origin based on the volatile fraction. Differentiation of EVOOs and VOOs is a project studied by many researchers, who have concluded that, among others, certain molecules such as monoterpenes, sesquiterpenes and terpenes are characteristic compounds that allow the effective classification of the studied samples [57,58,60]. As is mentioned in this review, the volatile profile of an olive oil, as defined by SPME combined with the gas chromatography technique, has been proven to be a valuable tool to determine authenticity. The majority of the mentioned articles discriminate olive oil according to its geographical origin. The fiber used by most researchers as the most adequate to collect volatiles was the 50/30 µm Divinylbenzene/Carboxen/Polydimethylsiloxane fiber. The extraction temperature of volatiles varied from 25 to 70 °C, while the incubation time varied from 5 min to 2 h. Extraction time also presented a great variability between the studies, from 5 to 90 min. Quite all studies used a chemometric method to explain results, most common of which were PCA and PLS analysis. Until now, the geographic origin of olive oil is verified by documented traceability supported by the stable isotope characterization as an official method to confirm the documented information provided by the producers. However, an important question that still needs an answer is how the geographic origin of an unknown sample is guaranteed using this method. Thus, the need for a method able to reliably verify the geographic origin of both known and unknown samples of olive oil is an urgent requirement. Literature data demonstrate that gas chromatography is a popular method to use when geographic origin should be declared. Olive oil is a complex mixture; however, as demonstrated by the above-mentioned studies, chemical interpretation of this mixture is achievable through gas chromatography. Consequently, a vast amount of data that describe the chromatographic profile of the examined olive oil are available, which, in combination with chemometric methods that point out differences between the studied samples, provide valuable information. In this regard, gas chromatography is a suitable method to use when the geographic origin of olive oil is required.

In addition, Infrared and Raman spectroscopy consist of another analytical, quick technique to consider when authenticity must be declared. Both methods deal mostly with the detection of possible adulteration of EVOO and VOO with minor quality or vegetable oils. According to the articles presented in this review, adulteration with sunflower oil is the most studied, followed by soyabean oil. The range of the concentration that the adulterant was detected was from 1 to 80%; however, this depends on the type of the adulterant. PLS and PCA were the most common chemometric methods used in combination with various algorithms. Although the number of studies that use IR spectroscopy to estimate authentication of olive oil is greater than those that use only Raman spectroscopy, results obtained from these studies showed that adulteration was successfully detected using both techniques based on spectral differences between olive oil of best and minor quality or olive oil and vegetable ones. Consequently, it cannot with certainty be estimated whether, for example, IR outweighs Raman spectroscopy.

In addition, both techniques allow the analysis of a large sample size within a day, while at the same time, no chemical waste is produced. Analysis can be qualitative and/or quantitative, and the information obtained is based on the presence of characteristic chemical groups that belong to specific chemical families. EVOO and VOO authenticity via IR and Raman spectroscopy is mainly based on their predominant constituents, namely, fatty acids and triglycerides. However, the determination of these constituents can also be achieved by gas chromatography. Although GC allows precise chemical characterization of the FA and triglycerides presented, it is a time-consuming method that requires sample pre-treatment with the use of non-environmentally friendly solvents. IR and Raman spectroscopy have overcome both these disadvantages which are of major importance, especially when rapid certification of authenticity is required. Nevertheless, GC may provide data that complete the fatty acid profile of an olive oil.

Now, the inverse situation is to be explained regarding the volatile profile. As is mentioned above, volatiles are among those compounds according to which authenticity can be determined. Emphasis was given to the chemical characterization and quantification of these compounds via gas chromatography, which, as indicated by literature data, is a popular method adopted by many researchers in pursuit of olive oil authentication. In the study of Azizian et al., 2015 [7], it was demonstrated that volatile compounds could be detected at the band near 5280 cm^−1^, attributed to carbonyl groups, using FT-NIR spectroscopy. A high-intensity band appears in the case of EVOO, contrary to other oils such as palm and refined oils. The intensity of the band is also gradually diminished during refining and deodorization processes and during storage. In this case, further analysis of the volatile profile of the olive oil is necessary due to the presence or absence of specific compounds that determine its authenticity.

Generally speaking, the techniques included in this review can be supplementary to each other since information for the presence of specific groups followed by their chemical characterization provides reliable, complete results of olive oil quality.

Obviously, multivariate methods proved to be the key tool that helped to cope with the complicated, large amount of data and to provide accurate results for quality control of the studied samples. Chemometrics is the discipline that combines mathematical and statistical methods to extract and understand all the information provided by a complex matrix of data. Complex data sets obtained are subjected to multivariate analysis, which examines the relationship between the different variables. Usually, a pre-processing step of the data is required (such as baseline correction, normalization of the data, noise suppression) in order to remove the information which does not contribute to data analysis and then the most suitable method is chosen based on the type of data to analyze and on the number of the predicted variables. The output may be qualitative and/or quantitative. In the first case, classification models are built, and the question to answer is whether a sample belongs to a specific group or not. In the second case, a prediction model is constructed, and the question now focuses on predicting an unknown concentration. Chemometric methods, in combination with various statistical methods such as PCA, PLS, SLRA, SLDA, CA and mathematical procedures, have been characterized as valuable data process tools for accurate characterization of olive oil [69,70,71,72]. However, experimental design when applying chemometrics is of major importance. The most appropriate models to need proper investigation; the selection of factors that contribute to the output and lead to optimal results should imply the use of the most adequate algorithm. Therefore, the use of chemometrics, although most of the time clarifies the relationship between the obtained data, requires a considerable amount of time to choose between a plethora of available models and algorithms.

Nevertheless, an important annotation that should not be neglected is that the mentioned studies use a control sample, usually an unadulterated one, in order to compare samples with known impurities. This may be considered as a serious drawback because in commercial EVOO and VOO, the degree and type of the adulterant is unknown. No example models are available, which could give the necessary information that enables researchers to confirm their results. Thus, the lack of a database that will provide information based on seasonal, cultivar, varietal parameters and on chemical information regarding possible adulterants to be used worldwide in order to validate and assert the obtained analysis results is evident. In addition, a new approach should be built regarding the use of analytical techniques by those organizations that impose the rules regarding olive oil authentication. Until now, only gas and liquid chromatography and ultraviolet spectroscopy have been adopted as official methods to guarantee authenticity. In addition to this, no worldwide instructions have been proposed regarding the olive oil market trade. Thus, in our opinion, not only should worldwide instructions be adopted imposing specific limits and guidelines that evaluate olive oil quality, but approval of more analytical techniques to be officially used is needed. Moreover, chemometrics may also be adopted by official regulators as a method for interpreting results, and chemometric models should be proposed to facilitate and harmonize analysis.

## 5. Conclusions

The review focuses on vibrational spectroscopic techniques, namely, infrared and Raman spectroscopy which are widely used to detect and quantify the adulterant based on characteristic absorption bands and gas chromatography, in which the volatile profile of an olive oil is considered one of the parameters that allow classification according to its geographical and botanical origin.

Although olive oil is under the strict quality control by official authorities, its authentication still remains a relevant problem since cases of fraud are not eliminated. Despite the fact that some analytical techniques have been adopted and characterized as official methods to confirm authentication, still, much effort should be given to reviewing these methods regarding their simplicity and efficiency. Furthermore, considering new methods such as IR and Raman spectroscopy, which are less time-consuming, environmentally friendly, accurate and cost-effective should be an issue for discussion among authorities.

## Figures and Tables

**Table 1:** Infrared and Raman spectroscopic methods used to evaluate authenticity of EVOO and VOO.

Authors	Method/Sample Preparation	Focus	Chemometrics
**Detection of Adulteration**			
[7]	FT-NIR spectroscopy/the method requires no sample preparation	Authenticity and adulteration of EVOO with vegetable oils	PLS1 analysis
[10]	FT-NIR spectroscopy/the method requires no sample preparation	Screening of commercial oils labeled as EVOOs to identify their authenticity	PCA and PLS analysis
[16]	NIR Raman spectroscopy/the method requires no sample preparation	Adulteration with hazelnut oil as well as botanical and geographical discrimination of EVOO	PLS and PCA analysis
[17]	FT-NIR Raman spectroscopy/the method requires no sample preparation	Geographical discrimination and adulteration of EVOO with sunflower oil	PCA analysis
[18]	Vis-Raman/the method requires no sample preparation	Quantitative determination of EVOO adulteration with sunflower oil	PLS and PCA analysis
[19]	Raman and visible spectroscopy/ both methods require no sample preparation	Detection of Greek EVOO adulteration with sunflower oil	PLS method
[20]	Raman spectroscopy/the method requires no sample preparation	Detection of adulteration of EVOO with soybean oil	PCA and LDA analysis
[21]	FT-IR, FT-NIR and Raman spectroscopy/transesterification of the sample	Discrimination of pure olive oil from adulterated olive oil and quantification of the adulterant	PCA, PLS-DA analysis
[22]	FT-IR/the method requires no sample preparation	Detection of adulteration EVOO with hazelnut oil	DA and PLS
[23]	FT-IR/the method requires no sample preparation	Detection of EVOO adulteration with canola, hazelnut, pomace and high linoleic/oleic sunflower oils	PLS analysis
[24]	FT-IR/the method requires no sample preparation/ for the quantification of the adulterant; in EVOO and palm oil mixtures, chloroform was added	Detection of EVOO adulteration with palm oil	PLS and PCR analysis
[25]	Mid-IR spectroscopy/the method requires no sample preparation	Detection of adulterated EVOO with sunflower and soybean oil	PLS and PLS-DA analysis
[26]	MIR-spectroscopy/the method requires no sample preparation	Adulteration of EVOO with lampante virgin oil	DA, PLS and PCA analysis.
[27]	NIR-spectroscopy/the method requires no sample preparation	Detection of EVOO adulteration with corn oil, sunflower oil, soybean oil and canola oil	PCA analysis
[28]	NIR-spectroscopy/the method requires no sample preparation	Geographic classification of Argentinian EVOO and detection of adulteration with sunflower, corn and soybean oils	PCA and PLS1-DA analysis
[29]	FT-NIR spectroscopy/the method requires no sample preparation	Adulteration of EVOO with peanut, sunflower seed oil, soybean, sesame and corn oil	PLS analysis
[30]	FT-NIR, FT-MIR and Raman spectroscopy/the method requires no sample preparation	Adulteration of EVOO with soyabean oil	PLS
[31]	MIR and NIR spectroscopy/the method requires no sample preparation	Adulteration of EVOO with refined and deodorized oils	PLS analysis
[32]	FT-NIR spectroscopy/the method requires no sample preparation	Adulteration of EVOO with sunflower, soybean, corn and refined oil	CI and SIMCA model
[33]	NIR spectroscopy/the method requires no sample preparation	Discrimination of EVOO from lower-quality oils (refined, pomace olive oil)	PCA and SIMCA classification model
[34]	ATR-FTIR spectroscopy/the method requires no sample preparation	Adulteration of EVOO with sunflower oil	DA, PLS analysis
[35]	ATR spectroscopy/the method requires no sample preparation	Adulteration of EVOO with refined soybean oil, refined sunflower oil, refined corn oil and refined canola oil	PLS analysis
**Discrimination According to Geographical-Varietal/Botanical Origin**			
[36]	Resonant Raman and isotope ratio mass spectroscopy/the method requires no sample preparation	Geographical discrimination of EVOO	LDA analysis
[37]	MIR-spectroscopy/the method requires no sample preparation	Geographic classification of Moroccan EVOOs	PCA and PLS-DA analysis
[38]	MIR spectroscopy/the method requires no sample preparation	Classification of VOO according to their geographical origin	PLS-DA analysis
[39]	NIR-spectroscopy/the method requires no sample preparation	Geographic classification of EVOOs from Eastern Mediterranean	FDA and k-NN method
[40]	Raman spectroscopy/the method requires no sample preparation	Qualitative and quantitate analysis of Andalusian EVOO	PLS-DA analysis
[41]	NIR spectroscopy/the method requires no sample preparation	Discrimination of VOOs according to their variety	PCA and SIMCA modeling
[42]	NIR and MIR spectroscopy/the method requires no sample preparation	Discrimination of EVOOs according to their variety	LDA analysis in combination with SIMCA model
[43]	ATR-FTIR spectroscopy/the method requires no sample preparation	Geographical discrimination of EVOOs	PLS and PCA analysis
[44]	ATR-FTIR spectroscopy/the method requires no sample preparation	Botanical discrimination of Greek EVOOs	Stepwise LDA analysis and QDA models
[45]	FT-IR spectroscopy/the method requires no sample preparation	Geographical discrimination of EVOOs	PLS/LDA and GA/LDA analysis

**Table 2:** Gas chromatography and volatile profile to evaluate authenticity of EVOO and VOO.

Authors	Method	Collection of Volatiles (e.T; i.t;e.t) *	SPME Fiber Type	Focus	Chemometrics
**Detection of Adulteration**					
[47]	SPME-MDGC	70 °C - 5 min	PDMS/DVB	Detection of adulteration of EVOO with hazelnut oil (based on filbertone) derived from unroasted hazelnuts	-
[48]	SPME/GC-MS	50 °C - 15 min	DVB/CAR/PDMS	Adulteration of EVOO with hazelnut oil	PCA analysis
**Discrimination According to Geographic Origin**					
[49]	GC-ITMS/SPME	40 °C 5 min 15 min	DVB/CAR/PDMS	Geographical discrimination of EVOO	PLS and LDA analysis
[50]	GC-SPME	40 °C 10 min 40 min	DVB/CAR/PDMS	Geographical discrimination of VOOs based on phenols and volatile compounds	Anova and PCA analysis
[51]	SPME/GC–MS	40 °C 5 min 60 min	DVB/CAR/PDMS	Volatile compounds as markers for the geographical differentiation of Spanish EVOO	PCA, LDA and SLDA statistical models
[52]	HS-SPME-GC-MS	70 °C 10 min 60 min	DVB/CAR/PDMS	Geographic authentication of EVOO based on sesquiterpenes profile	PCA, PLS-DA analysis.
[53]	HS-SPME/GC-FID and GC-MS Analysis	40 °C - 30 min	CAR/PDMS	Geographical characterization of Italian VOO based on volatile profile	ANOVA, DA, PCA analysis
[54]	HS-SPME, GC-MS and GC-FID	25 °C 30 min 50 min	PDMS	Characterization of virgin olive oil according to their geographical origin	ANOVA, PCA and HCA analysis
[55]	HS-SPME/GC–FID	25 °C 60 min 90 min	DVB/CAR/PDMS	Authenticity of VOO (labeled as PDO) with commercial EVOO	PCA analysis and SIMCA classification model
**Discrimination According to Variety/Botanical Origin**					
[56]	GCMS-SPME	40 °C 2 h 30 min	DVB/CAR/PDMS	Differentiation of Turkish EVOOs varieties	PCA analysis
[57]	HS-SPME-GC/MS GC-FID	50 °C 5 min 15 min	DVB/CAR/PDMS	Cultivar differentiation of Greek olive oil	MANOVA/LDA analysis
[58]	HS-SPME. GC × GC-TOF-MS 1D-GC–MS	50 °C 6 min 40 min	DVB/CAR/PDMS	Virgin olive oil differentiation according to variety and geographical origin	ANOVA, PCA and SLDA analysis
[59]	HS-SPME/GC-MS	50 °C 60 min 90 min	DVB/CAR/PDMS	Comparison of Algerian virgin olive oil in terms of variety and geographical origin	-
[60]	HS-SPME, GC-MS	25 °C - 50 min	DVB/CAR/PDMS	Geographical and varietal discrimination of EVOO based on monoterpene and sesquiterpene hydrocarbons profile	-
[61]	DHS-GC	40 °C - 15 min (sweep with N2)	-	Characterization of VOO varieties according to quantification of volatiles	SLDA analysis
[9]	SPME-GC–MS	25 °C - 15 min	DVB/CAR/PDMS	Triacylglycerols and volatiles of EVOOs different varieties	LDA analysis
[62]	HS-SPME-GC/MS	50 °C 5 min 15 min	DVB/CAR/PDMS	Cultivar and geographical classification of Greek olive oil	ANOVA analysis LDA
[44]	HS-SPME-GC/MS	50 °C 30 min 15 min	DVB/CAR/PDMS	Botanical discrimination of Greek EVOOs	stepwise LDA and QDA models

* e.T: extraction temperature; i.t: incubation time; e.t: extraction time.

## Abbreviations

ANN-MLP	Artificial Neural Network with Multilayer Perception
ANOVA	Analysis of Variance
BOSS	Bootstrapping soft shrinkage
CA	Cluster analysis
CAR/PDMS	Carboxen/Polydimethylsiloxane
CARS	Competitive adaptive reweighted sampling
CI	Univariate conformity index
DA	Discriminant analysis
DVB/CAR/PDMS	Divinylbenzene/Carboxen/Polydimethylsiloxane
EEC	European Community
EVOO	Extra virgin olive oil
FA	Fatty acid
FDA	Factorial discriminant analysis
FT-IR	Fourier transform infrared
GCXGC-TOFMS	Two-Dimensional Gas Chromatography and Time-of-Flight Mass Spectrometry
GC-FID	Gas chromatography with flame ionization detection
GC-ITMS	Gas chromatography-ion trap mass spectrometry
GC-MS	Gas chromatography combined with mass spectrometry
HCA	Hierarchical cluster analysis
HS-SPME	Headspace solid-phase microextraction
IOC	International olive oil council
IRIV	Iteratively retaining informative variables PLS
IRMS	Isotope ratio mass spectrometry
k-NN	K-Nearest Neighbor Analysis
LA	Linoleic acid
LDA	Linear discriminant analysis
LOX	Lipoxygenase
MADOVA	Multivariate Analysis of Variance
MCUVE	Monte Carlo uninformative variable elimination
MDGC	Multidimensional gas chromatography
MDS	Multidimensional scaling
MIR	Mid-infrared
MLR	Multiple linear regression
MUFAs	Monounsaturated fatty acids
NIRS	Near-infrared spectroscopy
NMR	Nuclear magnetic resonance
OO	Olive oil
PCA	Principal component analysis
PDMS/DVB	Polydimethylsiloxane/ Divinylbenzene
PDO	Protected Designation of Origin
PLS	Partial least squares regression
PLS-DA	Partial least square regression-Discriminant analysis
PLS-R	Partial least. Squares regression
POA	Palmitoleic acid
PUFAs	Polyunsaturated fatty acids
QDA	Quadratic discriminant analysis
RMSEC	Root means square error of calibration
RMSECV	Root Mean Square Error of Cross-Validation
RMSEP	Root Mean Squared Error of Prediction
RRS	Resonant Raman spectroscopy
SIMCA	Soft Dependent Modelling of Class Analogies
SAFA	Saturated fatty acids
SLDA	Stepwise linear discriminant analysis
SLRA	Stepwise linear regression analysis
SPME	Solid phage microextraction
SVM-C	Support vector machine using parameter C
VIP	Variable Importance of Projection
VOO	Virgin olive oil
